# Pulmonary fibrosis in a dog as a sequela of infection with Severe Acute Respiratory Syndrome Coronavirus 2? A case report

**DOI:** 10.1186/s12917-022-03191-x

**Published:** 2022-03-22

**Authors:** Barbara Colitti, Luca Manassero, Elena Colombino, Erica Ilaria Ferraris, Roberta Caccamo, Luigi Bertolotti, Alessio Bortolami, Francesco Bonfante, Valentina Papa, Giovanna Cenacchi, Fiorella Calabrese, Elena Bozzetta, Katia Varello, Maria Teresa Capucchio, Sergio Rosati

**Affiliations:** 1 Department of Veterinary Sciences, University of Turin, Turin, Italy; 2 Istituto Zooprofilattico Sperimentale Delle Venezie, Legnaro, Italy; 3 Department of Biomedical and Neuromotor Sciences, “Alma Mater” University of Bologna, Bologna, Italy; 4 Department of Cardiac, Thoracic, Vascular Sciences and Public Health, University of Padova Medical School, Padua, Italy; 5 Istituto zooprofilattico Sperimentale del Piemonte, Liguria e valle d’Aosta, Turin, Italy

**Keywords:** SARS-CoV-2, COVID-19, Dog, Pneumonia, Diagnosis

## Abstract

**Background:**

Interstitial lung disease is a heterogeneous group of conditions characterized by severe radiographic changes and clinicopathological findings. However, in the vast majority of cases, the cause remains unknown.

**Case description:**

In the present study, we reported the clinical case of a 3 years old female Bull Terrier presented in October 2020 to the Advanced Diagnostic Imaging Department of the Turin Veterinary Teaching Hospital with a progressive pulmonary illness characterized by dyspnea, exercise intolerance, and a diffuse and severe pulmonary interstitial pattern at imaging investigations.

Considering the clinical findings, the dog was included in a serological survey for Severe Acute Respiratory Syndrome Coronavirus 2 (SARS-CoV-2) infection in companion animals, showing positive results. Due to the further clinical worsening, the owners opted for euthanasia. At necroscopy, dog showed severe and chronic bronchopneumonia compatible with a Canine Idiopathic Pulmonary Fibrosis and with serological features linked to a SARS-CoV-2 infection.

**Conclusions:**

The comparison of these lesions with those reported in humans affected by Coronavirus Disease 2019 (COVID-19) supports the hypothesis that these findings may be attributable to the post-acute sequelae of SARS-CoV-2 infection in a dog with breed predisposition to Canine Idiopathic Pulmonary Fibrosis (CIPF), although direct evidence of SARS-CoV-2 by molecular or antigenic approaches remained unsolved.

**Supplementary Information:**

The online version contains supplementary material available at 10.1186/s12917-022-03191-x.

## Background

Spontaneous progressive pulmonary fibrosis of unknown cause, termed idiopathic pulmonary fibrosis (IPF), is a disorder that has been recognized in both humans and animals [1]. In the formers, IPF is characterized by specific clinical, physiological, morphological, and imaging features [2–4]. Although a great number of anecdotal accounts of a chronic respiratory condition in dogs, the disease is poorly characterized in this species, partly due to the difficulty of accurate diagnosis and the lack of accurate biopsy or postmortem material from these cases. The condition has been mainly documented in some terrier breeds, primarily in West Highland White and Scottish terriers and recently also in Staffordshire Bull Terriers [5–8]. Different potential known causes of lung disease have been advanced, such as infectious and parasitic agents, toxins and, immuno-mediated conditions [8, 9].

Susceptibility to SARS-CoV-2 infection has been demonstrated in different animal species under both natural and experimental infection [10–13]. In particular, since the first emergence in Wuhan, China, in December 2019, cases of SARS-CoV-2 infection in domestic animals housed with COVID-19 patients, have been increasingly reported worldwide [14–17], confirming a human-to-animal transmission of the virus. Up to date, outbreaks were notified to OIE in 10 animal species in 30 countries [18, 19]. Among dogs and cats, the former was shown to be less susceptible to SARS-CoV-2 infection [11, 13, 17, 20–23]. Moreover, in the great majority of reported cases animals were asymptomatic [10, 12] with a few exceptions of dogs and cats recovered for respiratory or gastroenteric symptoms [12, 14, 22, 24–26]. However, our knowledge on a comprehensive description of clinical and pathological outcomes in animals is missing.

## Case presentation

A 4-year-old, 27 kg, spayed female Bull Terrier was presented on November 3dr 2020 to the referent veterinarian for 2 weeks progressive fatigue after physical activity and increased respiratory rate at rest. Appetite was preserved and no other anomalies were found at the clinical visit.

Routine haematological and biochemical analyses revealed leukocytosis (17,84 x10E03/mcL; range 5,05-16,76) with raised monocytes (1,45 x10E03/mcL; range 0,16-1,12), raised ALP (144 U/I;range 16–119), GPT (80 U/I; range 22–78), total bilirubine (0,52 mg/dl; range 0,0-0,45), cholesterol (385 mg/dl; range 156–369), triglycerides (385 mg/dl; range 30–112) and total protein (8 g/dl; range 5,7-7,8).

**Fig. 1:**
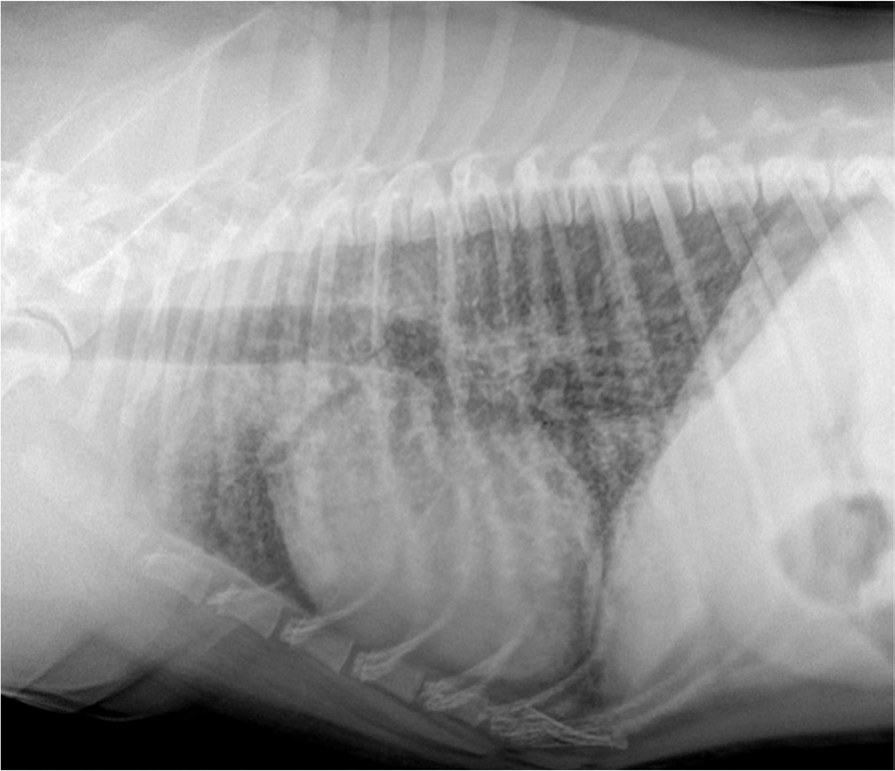
Thoracic radiograph showing a diffuse and severe pulmonary interstitial pattern

Thoracic radiographs showed a diffuse and severe pulmonary interstitial pattern (Fig. 1).

The dog was treated with antibiotics and non-steroidal anti-inflammatory (NSAIDs) drugs without improvements in symptoms by referring veterinarian.

One week later (November 10th) the patient was referred to the Advanced Diagnostic Imaging Department of the Turin Veterinary Teaching Hospital for further diagnostic investigations.

**Fig. 2:**
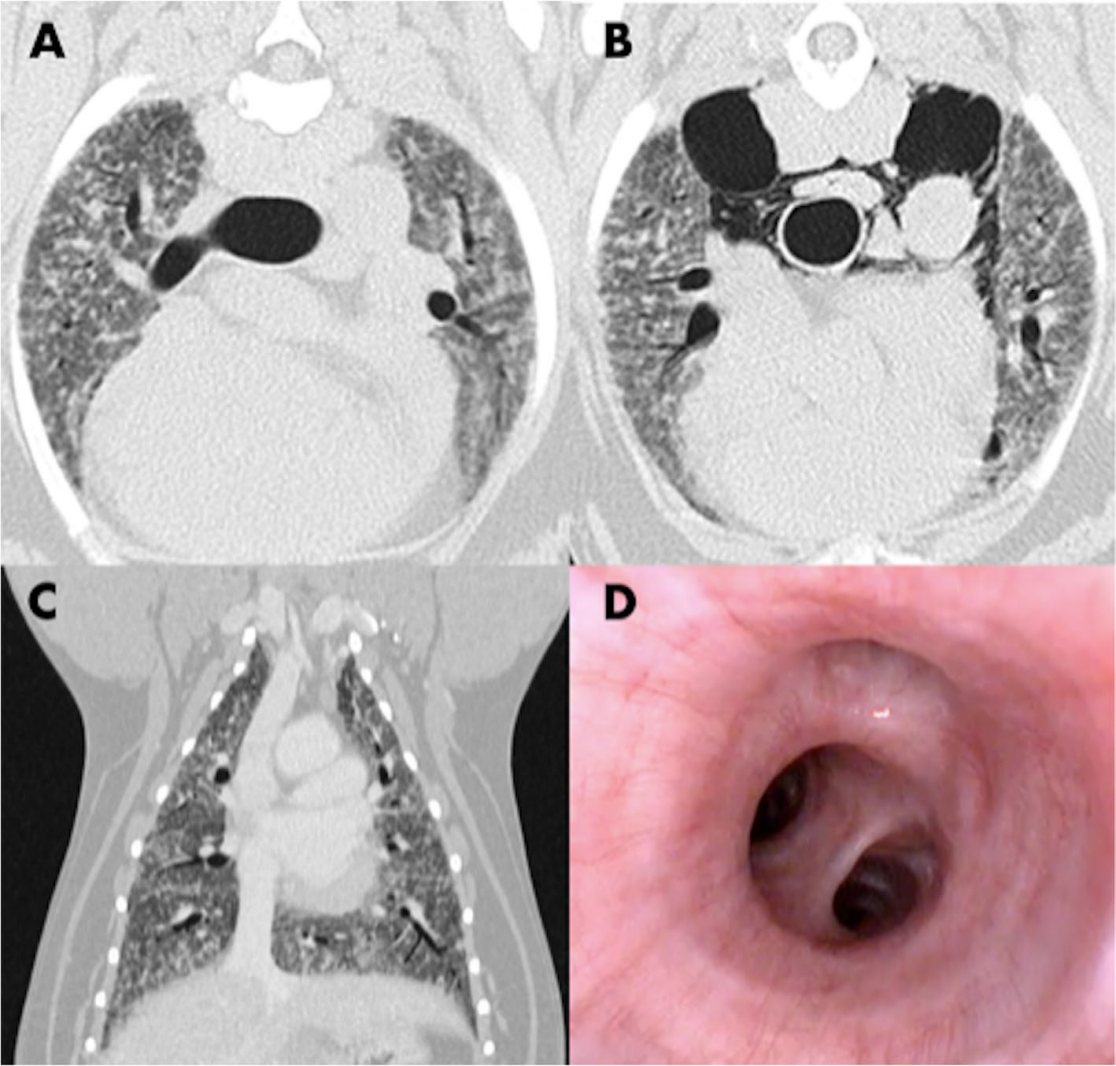
Diagnostic Imaging showing a diffuse interstitial thickening with parenchymal bands and reticular pattern in association with multiple and peripheral focal ground glass opacities < 1 cm in diameters. Computed tomography of thorax at presentation (**A**) and control after 2 months with onset of pneumomediastinum (**B**). MPR image showing the extension of the process in all pulmonary parenchyma (**C**). (**D**) Tracheo-bronchoscopy revealing segmental and sub-segmental bronchi of the right caudal lobe, showing normal conformation and mild mucosal edema and hyperemia

Total body Computed Tomography (CT) revealed a diffuse interstitial thickening with parenchymal bands and a diffuse reticular pattern in lungs. Multiple and peripheral focal ground-glass opacities were also found as small hyperattenuating areas of < 1 cm in diameters. No other abnormalities were found in vascular and bronchial structures (Fig. 2, A). Given the stable conditions of the patient, even though fatigue and mild tachypnea persisted, no therapy was set.

Four weeks later (December 7th) a tomographic control was performed, revealing a mild worsening of the pulmonary interstitial pattern, with the onset of moderate pneumomediastinum, probably due to a bilateral rupture of the lungs in the cranial lobes (Fig. 2, B).

A tracheo-bronchoscopy was also performed, in order to obtain a bronchoalveolar lavage (BAL). A first-degree tracheal collapse was found in the cranial portion. At the level of the tracheal bifurcation, a dorsoventral movement was clearly visible, but in the absence of signs of collapse. Left and right bronchial tree were apparently unaffected in terms of conformation, with mild mucosal edema and hyperemia. No exudate was found (Fig. 2, D).

BAL cytology showed a mild neutrophilic inflammation with extracellular bacteria and yeasts, but oropharyngeal contamination was not excluded.

The mycological examination revealed the presence of colonies with a yeast-like appearance, compatible with *Trichosporon* and *Geotrichum* genera. They were considered as contaminants not related to the pathological process since they were detected outside the neutrophils on cytological examination. Symptomatic therapy with Prednicortone (0.5 mg/kg) was set.

Due to the severe interstitial pneumonia, the dog was included in a serological survey conducted in Italy to assess the prevalence of antibodies against SARS-CoV-2 Nucleoprotein (Np) in domestic animals [27, 28].

**Fig. 3:**
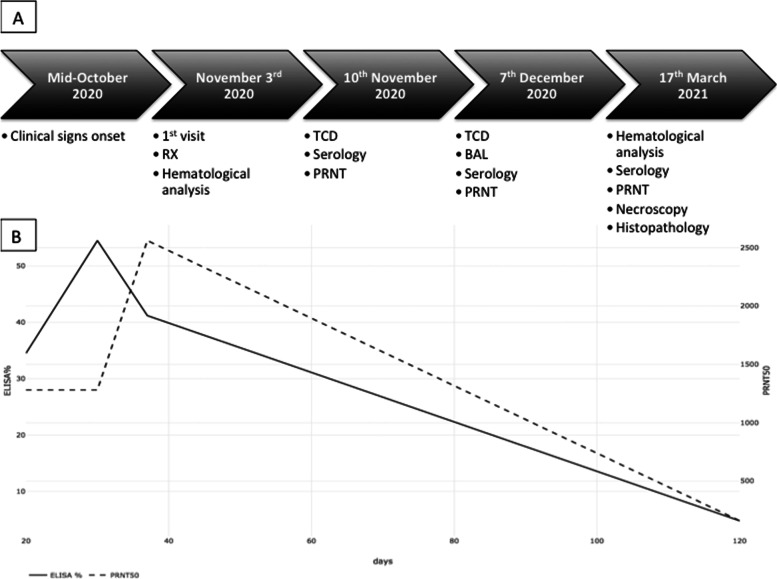
**A** Case report timeline. **B** Scatter plot comparing the results of the Double antigen ELISA test and PRNT50 test at the time of serum sample collection

Serum samples were collected on days 32, 42, 49, and 4 months after the putative estimated onset of clinical signs. The complete timeline of diagnostic investigations performed is summarized in Fig. 3A. Nasopharyngeal and rectal swabs were also collected at days 32 and 49 and immediately stored at − 80 °C.

Three months later (March 2021), the patient came back to the Emergency Care Unit of Turin Veterinary Teaching Hospital for a severe dyspnea, refractory to antibiotics and cortisone, after a gradual worsening of her condition during the last month.

At the examination, the patient was still alert but showed severe dyspnea and inability to rest. The patient was hospitalized, and oxygen therapy was administered in a double nasal tube and medical therapy had been set up (Butorphanol 0,2 mg/kg IV and Marbofloxacin 2 mg/kg PO SID). It was agreed with the owner to detain the patient for further diagnostic investigations.

Haematological analyses revealed leukocytosis (WBC: 50,18 cells/mcL; range 5,2–17,9) with neutrophilia (39,14x10E03 cells/mcL; range 2,9-12,5) and monocytosis (6,52x10E03 cells/mcL; range 0,2-1,2). Reticulocytosis was also present (163,2x10E03 cells/mcL; range 8,4-129,3), with ghost cells and polychromasia.

Due to the further worsening of dyspnea, the owners opted for euthanasia. Necropsy was performed according to standard procedures, and the following tissue samples biopsies were collected in sterile tubes and 10% neutral buffered formalin-fixed: heart, liver, kidney, spleen, stomach, small intestine, large intestine (colon), lung, trachea, cranial mediastinal lymph nodes, and brain. The tissues were routinely processed for histopathological examination and Periodic Acid Schiff (PAS) and Masson’s Trichrome stainings were made on lung sections to exclude the presence of fungi and to better evaluate the increase in connective tissue respectively. Immunohistochemistry for SARS-CoV-2 was also performed on selected lung sections. Immunohistochemical analysis was performed using the monoclonal antibody anti-SARS-CoV-2 Nucleocapsid (Sino Biological) at dilution 1:2500 and the polyclonal anti-SARS-CoV spike glycoprotein (S) (Sino Biological) at dilution 1:4000. The sections were viewed using Axio Scope 5 microscope equipped with Axiocam 105 color and images were acquired with 10x objective using ZEN lite software (Zeiss, Jena, Germany).

Selected paraffin-embedded samples of lung and kidney were also submitted to ultrastructural investigations. Briefly, samples were retrieved from paraffin blocks, brought to water, and put in cacodylate buffer before being post-fixed in 1% osmium tetroxide, dehydrated in alcohol, and embedded in Araldite. Ultrathin sections were stained with uranyl acetate, lead citrate and examined with a Philips TEM CM 100 transmission electron microscope (Philips, Amsterdam, The Netherlands). Histological images of representative fields from lungs were captured with a Nikon DS-Fi1 digital camera coupled to a Zeiss Axiophot microscope using a 20× and a 2.5x objective lens. NIS-Elements F software was used for image capturing at a resolution of 150 dots x inch (dpi). An image processing program (Luminar 4, Skylum, USA) was then used to enhance the resolution of the image to 300 dpi.

Blood serum samples were tested using a recombinant double antigen SARS-CoV-2 N ELISA (In3diagnostic, Torino, Italy) and a Plaque Reduction Neutralization Test (PRNT) as previously described [29]. In order to rule out a potential aspecific result due to cross-reaction with Canine Respiratory and Canine Enteric Coronavirus, sera were also tested against these related coronaviruses antigens as previously described [28].

Viral RNA was extracted from swabs, BAL, and post-mortem tissue biopsy samples using Qiamp Viral Mini kit (Qiagen, Hilden, Germany) and subjected to RT-qPCR targeting SARS-CoV-2 RdRP gene [30]. Moreover, 10 μl of eluted RNA were reverse-transcribed using Maxima H-Minus dsDNA synthesis kit (ThermoFisher Scientific) following manufacturer’s instructions. Illumina libraries were prepared using Nextera XT DNA Library Preparation kit (Illumina, San Diego, CA, USA), according to the manufacturer protocol. The library concentration and quality were evaluated with fluorimetric method Qubit High Sensitive dsDNA kit (Life Technologies). Libraries were sequenced on MiSeq platform with the V2 500 cycles chemistry. Raw sequencing reads were analyzed as previously described [31].

Blood sera gave positive results in all but the last sample in double antigen Enzyme Linked Immunosorbent Assay (ELISA) and in all samples by PRNT tests, confirming a seroconversion against SARS-CoV-2 Np and spike protein, respectively. A decrease in antibodies titer was detected between the third and the last blood sample with both tests (Fig. 3B).

Negative results were obtained with both RT-qPCR and next generation sequencing approaches. Moreover, no sequencing reads belonging to other canine bacteria or viruses were found.

**Fig. 4:**
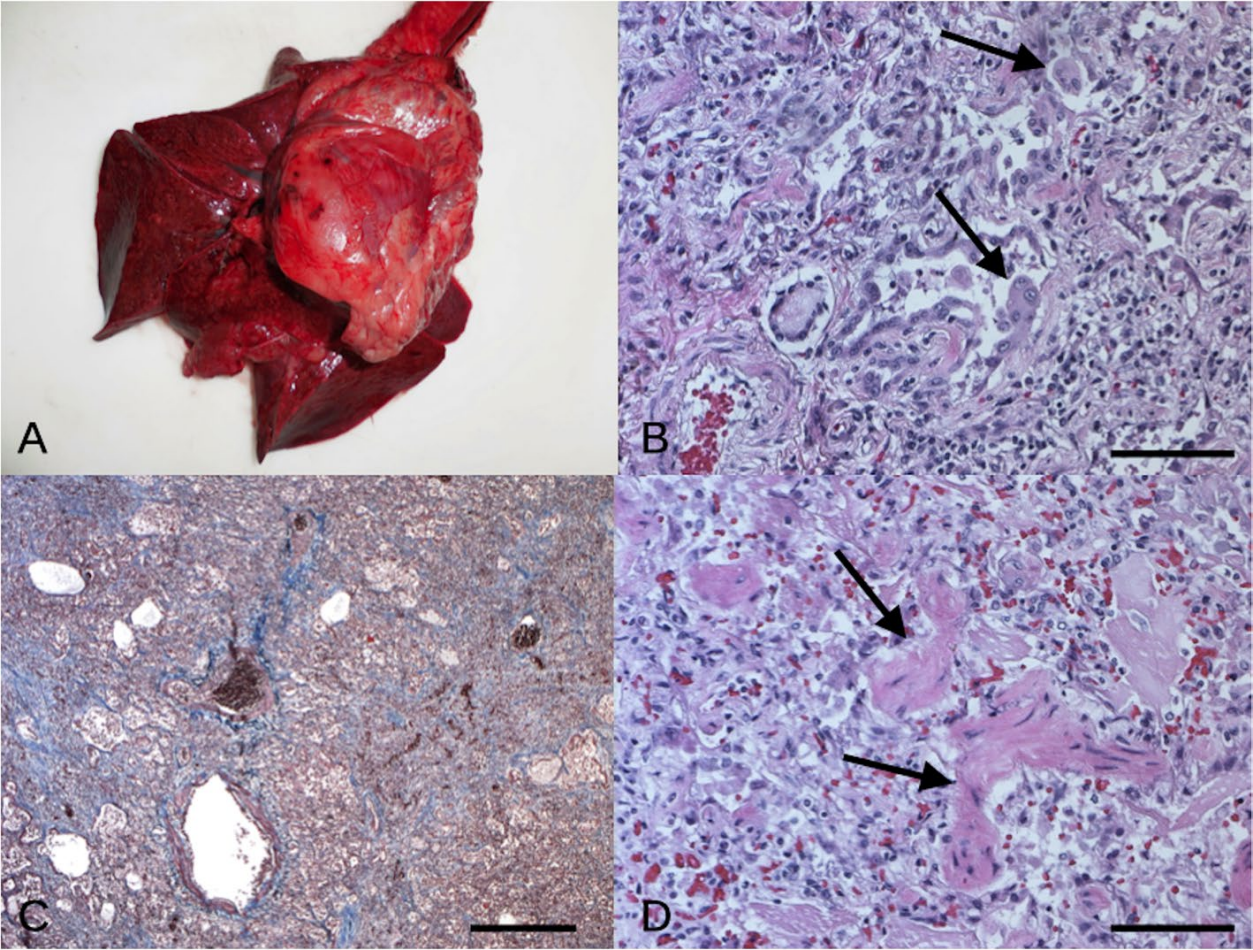
Dog. **A** Lung and heart. Diffuse and severe pulmonary consolidation (chronic broncho-pneumonia) and hypertrophic cardiomyopathy with focal epicardial petechiae. **B** Lung, pneumocyte hyperplasia/metaplasia (black arrow) and lymphoplasmacytic interstitial pneumonia, Haematoxylin and eosin (H-e), 20x, (scale bar = 50 μm). **C** Lung, severe and diffuse interstitial fibrosis (blue areas), Masson’s Trichrome staining, 2.5x, (scale bar = 200 μm). **D** Lung, moderate hyperplasia of the smooth muscle cells, H-e, 20x, (scale bar = 50 μm)

At necropsy, the dog was in a good state of nutrition (Body condition score: 4/5). In the abdomen there was moderate hepatomegaly and a pale, normal in size spleen. Moreover, a mild multifocal acute gastritis and a diffuse thickening of the intestine wall were recorded. At the thorax opening, a severe pneumomediastinum was observed. Lungs were dark-red colored and they showed a severe and diffuse pulmonary consolidation with miliary to coalescent nodular hyperplasia (Fig. 4A). Cranial mediastinal lymph nodes were hyperemic but normal in size. The heart showed severe hypertrophic cardiomyopathy with focal epicardial petechiae (Fig. 4A). All the other organs did not show any significant alteration.

At histological examination, the liver showed severe and diffuse vacuolar degeneration of the hepatocytes, mainly in periportal areas. Spleen had a severe and diffuse depletion of the red pulp. Regarding the gastrointestinal tract, the stomach presented a mild multifocal gastritis with multifocal lymphoid hyperplasia in the submucosa while mild to moderate lymphoplasmacytic enteritis with multifocal lymphoid hyperplasia was recorded in the small intestine (proximal portion of the duodenum). Kidneys showed bilateral moderate and multifocal membranous glomerulopathy with moderate hyperemia and hemorrhages. Lungs presented severe and diffuse edema and congestion with chronic interstitial broncho-pneumonia characterized by endoalveolar hyaline membranes and pneumocyte hyperplasia/metaplasia with multinucleated giant cell formation (Fig. 4B). Alveolar septa were enlarged by severe and diffuse lymphoplasmacytic infiltrates (monocytes, macrophages and lymphocytes) and fibrosis, confirmed by Masson’s Trichrome staining (Fig. 4C). Moderate hyperplasia of the smooth muscle cells around the terminal bronchioles was also recorded (Fig. 4D). Neoangiogenesis was also present, mainly subpleural. Also, multiple pulmonary intra-parenchymal micro-calcifications, hemorrhages, and hyperemia were observed. PAS staining was negative excluding a fungal infection.

Immunohistochemistry on lung resulted negative for both antibodies. A disseminated anthracosis with moderate hyperemia was recorded in the cranial mediastinal lymph nodes. The heart presented moderate and diffuse hypertrophy of the myocytes both in the right and left ventricles. Large intestine, trachea and, brain did not show any significant lesions. At ultrastructural examination, no viral particles were detected in the lung (Supplementary Figure). Multifocal to disseminated membranous glomerulopathy was detected in the kidneys, characterized by alterations of the glomerular basal membrane and electron-dense deposits.

## Discussion and conclusions

Spontaneous progressive pulmonary fibrosis of unknown cause is a poorly documented disorder of dogs, characterized by a progressive and devastating interstitial lung disease leading to clinical manifestation of dyspnea, coughing, exercise intolerance, and final respiratory failure. A breed predisposition in terriers is well documented, although the specific aetiopathogenesis remains unknown. Infectious diseases, hypersensitivity reactions, immune complexes, and toxins have been postulated as possible triggers [8, 32].

Also, since the first emergence in Wuhan in December 2019, SARS-CoV-2 infection was confirmed in an increasing number of animals, in particular dogs and cats living in close contact with COVID-19 positive owners [15, 33, 34].

The dog described in this study showed clinical, imaging, and histopathological features compatible with CIPF of unknown origin. Moreover, the entire clinical presentation resembled a SARS-CoV-2 infection as previously reported [12]. In fact, fatigue and breathing distress along with the presence of ground-glass opacities in lungs observed in thoracic radiographs were recorded also in human patients with symptomatic infection by SARS-CoV-2 [35]. Moreover, histopathological findings of interstitial pneumonia with pneumocyte hyperplasia, focal lymphocytic inflammation, multinucleated giant cell formation with hyaline membranes were compatible with diffuse alveolar damage in the proliferative phase, observed during viral pneumopathy due to SARS-CoV-2 infection [36]. Other commonly recognized causes of interstitial lung disease, such as canine infectious diseases, parasitic agents, toxins, cardiogenic and non-cardiogenic pulmonary edema, or infiltrative neoplasia were discarded. Many of these conditions have, in fact, rapid clinical onset and response to treatment in contrast with the case presented in this report, and no traces of known pathogens have been detected with the *Denovo* sequencing approach. It is noteworthy that Staffordshire bull terriers also seemed to be predisposed to chronic idiopathic pulmonary fibrosis, which is characterized histopathologically by two different patterns of interstitial fibrosis, one mature and the other immature [8]. Due to the fact that CIPF is characterized by non-specific inflammation and fibrosis of the pulmonary interstitium and peripheral airspaces and can be triggered by chronic viral infection [8], higher susceptibility to SARS-CoV-2 infection due to CIPF cannot be excluded in the dog of the present study. Moreover, cardiac involvement in severe COVID- 19 pathogenesis has been well documented and the hypertrophic cardiomyopathy recorded in this study can be due to pulmonary hypertension, which is a well-known complication of interstitial lung disease [37, 38]. Furthermore, membranous glomerulopathy can be a consequence of SARS-CoV-2 infection, as evidenced by the fact that 30% of SARS-CoV-2 infected human patients developed diverse glomerular and tubular diseases due to direct viral infection or indirect effects on the renin-angiotensin-aldosterone system, hemodynamic instability, coagulopathy, and cytokine storm [39]. In the present study, no viral particles were detected at ultrastructural examination in kidney and lung. However, the absence of viral inclusions in lung and kidney has been already reported in many SARS-CoV-2 human cases [36].

Regarding other organs, severe and diffuse vacuolar degeneration in the liver, mild gastritis, and enteritis were probably iatrogenic due to NSAIDs and cortisone administration [40].

Nevertheless, the longitudinal approach allowed confirming an active seroconversion against SARS-CoV-2 and a decrease in antibody titers between the 32nd and the 120th day after the onset of clinical signs with both the double antigen ELISA and the highly specific PRNT tests, which recognize two different antigens of the virus, the nucleoprotein and the spike protein respectively. The evidence of antibody response, as detected by two independent assays, strongly suggests that the dog had been exposed to SARS-CoV-2, sometimes during October 2020. It is noteworthy that two out of three family members voluntarily underwent a serological test for COVID-19 with negative results and the source of infection remained unsolved. On the other hand, the absence of positive results on nucleic acids testing and immunohistochemistry poses the major limit for reaching a diagnosis of cause-and-effect relationship and may reflect yet another case of canine pulmonary fibrosis in which only an idiopathic component is the cause of the clinical symptoms recorded. However, this finding may be justified by the time elapsed between the onsets of symptoms and the first samples carried out, supported by the short-lived viral shedding in dogs infected with SARS-CoV-2 (2–6 days p.i) as reported in other longitudinal studies [13, 25, 26].

In conclusion, the present study reported a case of canine pulmonary fibrosis of unknown origin with serological positivity against SARS-CoV-2. The comparison between histopathological findings in the dog and humans affected with COVID-19 supports the hypothesis that the clinical outcome may be the post-acute sequelae of SARS-CoV-2 infection, although a definitive diagnosis was not possible. This study highlights the importance of reporting clinical symptoms and outcomes also in animals and suggests strengthening diagnostic methods to elucidate the impact that SARS-CoV-2 infection has on different species and (if any) breed susceptibility.

## Supplementary Information

**Additional file 1 Supplementary Figure.** Lung, negative immunostaining for monoclonal antibody anti-SARS-CoV-2 Nucleocapsid (IHC), 10x (scale bar = 50 μm).

## Data Availability

The Next generation sequencing dataset used during the study is available in the repository NCBI BioSample database (BioProject ID: PRJNA755149).

## Abbreviations

SARS-CoV-2: Severe Acute Respiratory Syndrome Coronavirus 2
COVID-19: Coronavirus Disease 2019
CIPF: Canine Idiopathic Pulmonary Fibrosis
IPF: Idiopathic pulmonary fibrosis
CT: Computed Tomography
BAL: Bronchoalveolar lavage
Np: Nucleoprotein
S: Spike glycoprotein
ELISA: Enzyme Linked Immunosorbent Assay
PRNT: Plaque Reduction Neutralization test
NSAIDs: Non-steroidal anti-inflammatory
