# Supplementary figures and images for: Pulmonary fibrosis in a dog as a sequela of infection with Severe Acute Respiratory Syndrome Coronavirus 2? A case report
Source: BMC Vet Res. 2022 Mar 22;18:111. doi: 10.1186/s12917-022-03191-x (PMC8938595; doi:10.1186/s12917-022-03191-x)

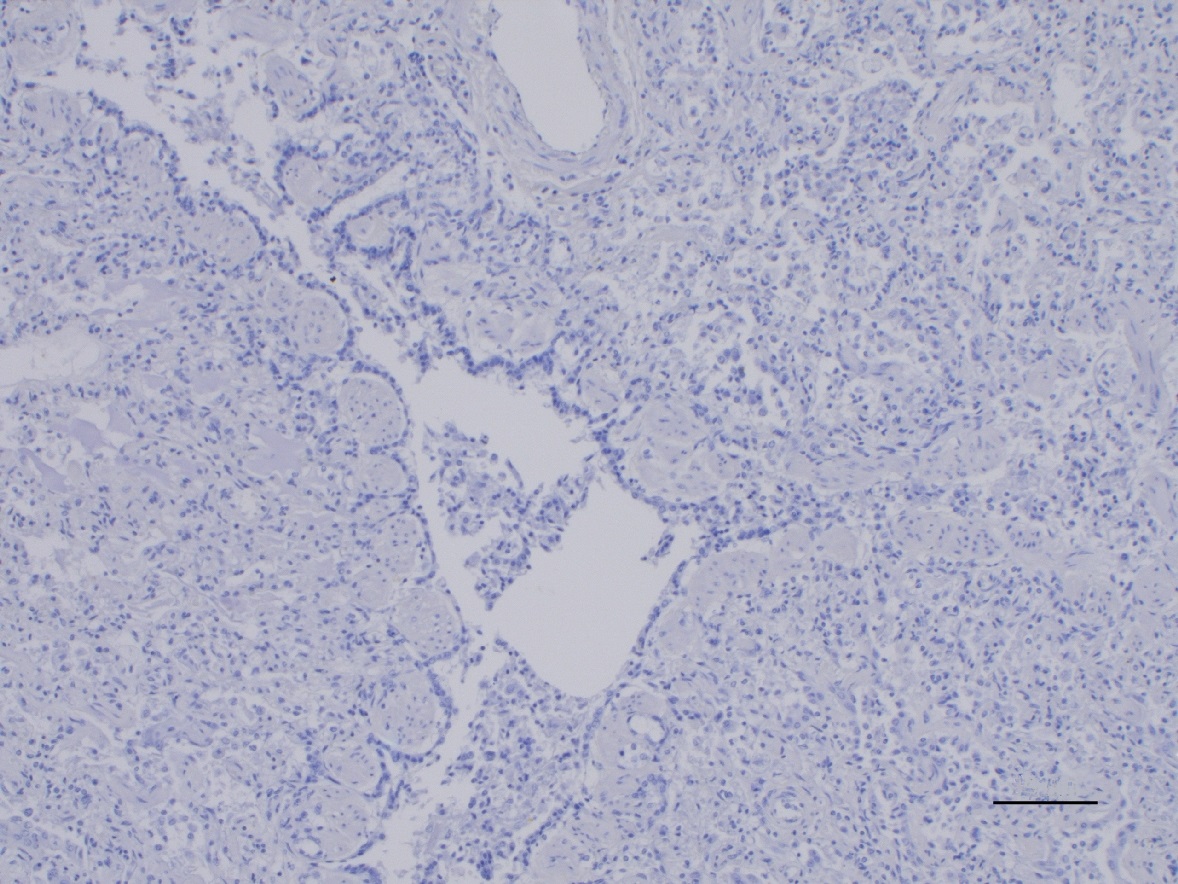

Supplement: Supplementary file 1 — Additional file 1 Supplementary Figure. Lung, negative immunostaining for monoclonal antibody anti-SARS-CoV-2 Nucleocapsid (IHC), 10x (scale bar = 50 μm). [file 12917_2022_3191_MOESM1_ESM.jpeg]
